# Characterization of Growth Secondary Hair in Min Pig Activated by Follicle Stem Cell Stimulated by Wnt and BMP Signaling Pathway

**DOI:** 10.3390/ani13071239

**Published:** 2023-04-03

**Authors:** Xinmiao He, Ziliang Qin, Ran Teng, Ming Tian, Wentao Wang, Yanzhong Feng, Heshu Chen, Haijuan He, Haifeng Zhang, Di Liu, Xinpeng Jiang

**Affiliations:** 1 Key Laboratory of Combining Farming and Animal Husbandry, Ministry of Agriculture and Rural Affairs, Animal Husbandry Research Institute, Heilongjiang Academy of Agricultural Sciences, No. 368 Xuefu Road, Harbin 150086, China; 2 College of Animal Science and Technology, Northeast Agricultural University, Harbin 150030, China

**Keywords:** Min pig, follicle stem cell, BMP, Wnt

## Abstract

**Simple Summary:**

The Min pig is a national-level protected pig that is recognized as a rare animal in China, lives in a constantly cold environment in the north of China, and has excellent characteristics compared with other commercial pigs. The Min pig has the specific character of secondary hair growth in winter and seasonal cycling. International research on hair follicles is mainly focused on human and mice hair, including hair follicle regeneration and hair loss treatment. Research on hair follicles in pigs is limited. The structure and morphology of hair follicles from Min pigs are different from those of lean-type pigs, such as Yorkshire and Berkshire pigs, which do not grow secondary hairs in the adult stage. In this study, there were significant gene expression differences with high-throughput sequencing to determine the primary and secondary hairs. Based on skin blocker experimental results, we can infer that the Wnt and BMP signaling could stimulate follicle stem cells in the Min pig.

**Abstract:**

In China, the national-level protected pig, the Min pig, is characterized by the development of secondary hairs and hair follicles in winter. Factors that dominate the genotype in the growth of secondary hairs are not clear through the concrete cell signaling pathways. This study compared hair phenotypes based on morphological structure, transcriptomics, and potential targeting molecules in the breeds of Min, Berkshire, and Yorkshire pigs. The results indicated that Min pigs have specific characteristics for the growth of secondary hairs compared with the Berkshire and Yorkshire pigs. The transcriptome analyses and quantitative reverse transcription-polymerase chain reaction results revealed that secondary hair growth was activated by follicle stem cells. The specific inhibitors of Wnt and BMP were studied using respective signals. The density of follicles, activity of follicle stem cells, and relative gene expression results have shown that Wnt and BMP stimulate the activity of follicle stem cells, and the Wnt signaling molecule has a significantly better effect than the BMP signaling molecule on stem cells. Wnt and BMP can promote the growth of local secondary hair and gene expression. Therefore, this study was conducted to verify the development mechanisms of secondary hairs, which have potential applications in laboratory animals and comparative medicine.

## 1. Introduction

Pigs are economically important in many countries worldwide, and pig breeding is a matter of biosecurity. Gene studies indicate that pigs in Asia and Europe have evolved independently of the wild boar subspecies [1]. Modern breeding practices have been intensively pursued commercially for better growth, meat quality, and fertility traits in pig breeds, which has resulted in the loss of high genetic diversity under less selection pressure and adaptation to the growing environments [2]. With the development of the genome sequencing technologies, the study and selection of genetic phenotype signatures among different breeds plays an important role in understanding the process of breed development and the maintenance of human health [3].

Min pig is a national-level protected pig that is recognized as a rare animal in China, and lives in a constantly cold environment in the north of China [4] and has excellent characteristics, such as good meat quality [5], high fertility, and strong resistance to adversity [4]. The Min pig has the specific character of secondary hair growth in winter and seasonal cycling [6]. The primary hair cycling of the adult pig has passed through one complete cycle per year with an annual cycle in domestic pigs. All these pig breeds, namely, Yorkshire, Berkshire, and Min pigs, grow the secondary hairs in the period of fetal porcine, but the first two lose them with growth into the adult stage whereas the Min pigs do not. Most of the follicles in adult pigs are active for a 4-month period during autumn and early winter. However, only 20% of the primary hair follicles are in anagen during the remaining period from winter to the end of summer [7]. The domestic pig has been found to have seasonal cycling of primary hair, which could re-grow in the next anagen. However, there is no research on how the adult pigs grow secondary hairs with the whole hair cycle. In comparison with other pig species, the characteristics of the secondary hairs and hair follicles of Min pigs are significantly different than the domestic pig, which are important factors contributing to the ability of Min pigs to adapt to an extremely cold environment [5,8]. Meanwhile, our team studied the unique villous growth of Min pigs through the determination of follicle phenotype and of hair follicles, and the acquisition of sequencing data through RNA-seq to analyze differentially expressed genes in different seasons [9]. The most important is that the Min pig grows new secondary hairs every cold season. Hair follicle growth follows a definite pattern, which probably depends on hair density and/or the mammalian group, despite the general parallels in hair follicle and hair fiber differentiation. 

The structure and morphology of hair follicles from Min pigs are different from those of lean-type pigs, such as the Yorkshire and Berkshire pigs, which do not grow secondary hairs in the adult stage. Animals usually have specific features, such as a thicker coat and secondary hairs, which reduce heat loss from the animal and cold skin irritation for their protection and functioning in cold environmental conditions [10,11]. All of these characteristics come from the evolutionary selection pressure to adapt to extreme living conditions, such as temperature and diet [12,13]. Increasing evidence has established an important relationship between secondary hair characteristics and marker-assisted selection for animals with secondary hairs, which correlated with a shorter calving interval in Morada Nova sheep [2].

At present, international research on hair follicles is mainly focused on human hair, including hair follicle regeneration and hair loss treatment [14]. Research on hair follicles in pigs is limited. The cell biology of pigs is closest to that of humans, though, and it is of great importance to study the growth mechanism of pig hair follicles, since such studies can assist in the development of treatments for human hair loss. The earliest research on pig skin was the development of fetal porcine skin from 41 days of gestation back to birth [15]. The growth of hair in domestic pigs was not affected by the breeding season. Most importantly, there was no research on the regeneration of secondary hairs in any other adult pig. Our team focused on the regeneration of secondary hairs for Min pigs in the cold season, and this study provides a crucial animal model for studying the development of secondary hair follicles. Moreover, it was not clear that the secondary hairs possess the best stem cell properties stimulated by various signaling pathways in the adult Min pigs. To this end, we studied the difference in hair morphology and structure in three pig breeds, namely, Min pig, Yorkshire pig, and Berkshire pig, using high-throughput sequencing to determine gene expression differences. Thus, the present study was conducted to evaluate the relationship between the signaling pathway of stem cells and the hair morphological structure in Min pigs and its contribution to the thermoregulatory mechanisms in hair regeneration and hair loss.

## 2. Materials and Methods 

### 2.1. Animals and Sample Preparation

The Min, Yorkshire, and Berkshire pigs were procured from the Experimental Base of the Animal Husbandry Institute, Heilongjiang Academy of Agricultural Sciences. This study was conducted following the animal welfare guidelines of the World Organization for Animal Health. All the pigs were killed under pentobarbital sodium anesthesia. All the clinical animal samples used in this study were approved by the Committee on Ethics of the Heilongjiang Academy of Agricultural Science. Nine 7-month-old pigs (male/female) were randomly selected from each species (Min pig, Yorkshire pig, and Berkshire pig). All 9 pigs weighed approximately 100 kg, and the body length was approximately 120 cm in the winter. There were 3 pigs per pen with an equal number of barrows and gilts. Pens were subjected to the same dietary treatments as the nutrient requirements of swine, and they were balanced based on pen weight at the start of the study. The feeding and management conditions were similar for all pigs. Skin samples (25 mm^2^) were obtained from the skin tissue of Min, Yorkshire, and Berkshire pigs at the hair follicle developmental stages. The Min pigs could only grow the secondary hair follicle in the cold season, and the Yorkshire and Berkshire pigs could not grow the secondary hair follicle in adult life. The telogen of the secondary hair follicle is from June to October, and the anagen is from December to May of the next year in Min pigs. Skin tissue from the front shoulder of each pig was selected. The hairs were extracted by surgical forceps from the skin, and the bottom of hairs were cut to collect the hair follicle for RNA extraction. We obtained 30 lateral primary hair samples and 30 secondary hair samples within each skin tissue as technical repeats in the hair follicle development stage. The primary hair samples and secondary hair were obtained from the Min pig. The Yorkshire and Berkshire pigs were obtained for primary hair samples, which did not grow the secondary hair. The samples of the secondary hair were instead of skin tissue. A total of hairs of Min, Yorkshire, and Berkshire pigs were obtained in each pig for this study [16,17]. Hair length was measured using a Vernier caliper. A stereomicroscope was used to observe and analyze the distribution and number of secondary and primary hairs in Min pigs. All samples were immediately frozen in liquid nitrogen. All the samples were performed for three replications, and all the groups were examined as independent measurements.

### 2.2. Histological Study of Hair Follicle and Skin with H&E Staining

Hair follicle and skin tissue samples were collected (2 × 2 cm) from Min, Berkshire, and Yorkshire pigs for histological analysis. The slices of skin from the scapula were fixed with 4% formaldehyde for 2 days. Thereafter, they were dehydrated using different concentrations of alcohol. Xylene was used twice to make the tissue of the skin transparent for approximately 1.5 h each time when the stove was transparent through light at 50 °C. The dehydrated tissues were then waxed for paraffin embedding. The slices were roasted briefly in a temperature box at 60 °C, and the paraffin in the tissue space was removed using xylene [18]. The slices were washed three times with phosphate-buffered saline (PBS, pH = 7.4) and 0.25% trypsin for antigen repair. The paraffin sections were stained with 1% hematoxylin and 0.5% eosin (H&E) for histological examination using a light microscope (Olympus, Tokyo, Japan). The dewaxed sections were used for immunofluorescence assay.

### 2.3. RNA Extraction

The hair follicles of the primary and secondary hair samples were collected by hair extraction; they were pulled out to add in the Trizol for the next step of RNA extraction. The hair follicles and skin tissues were ground in liquid nitrogen and mixed with 1 mL Trizol, followed by incubation for 2–5 min at room temperature (RT). Total RNA was extracted using Trizol reagent (Invitrogen, San Diego, CA, USA), treated with TURBO DNase I (Ambion, TX, USA) for 30 min, and purified using a 2100 Bioanalyzer Nanochi (Agilent Technologies, Palo Alto, CA, USA) according to the manufacturer′s instructions. The RNA was further treated with phenol-chloroform and washed twice. Subsequently, the RNA-containing supernatant was transferred to a new tube, followed by ethanol precipitation, washing, and dissolution in diethyl pyrocarbonate-treated water. For high-throughput sequencing, there were at least 50 hair follicular units in one sample and for the reverse transcription-polymerase chain reaction (qRT-PCR) experiment, there were at least 200 hair follicular units in one sample. 

### 2.4. RNA-Seq Experiments

TRIzol reagent (Invitrogen) was used to extract RNA from the hair follicles and skin tissues according to the manufacturer’s instructions. A 2100 Bioanalyzer Nanochip (Agilent Technologies) was used to determine RNA purity. The TruSeq RNA Sample Prep V2 kit (Illumina, San Diego, CA, USA) was used to construct libraries for sequencing, as previously mentioned [19]. Reverse transcriptase and random primers were added to the reverse transcription for the interrupted mRNA fragments to synthesize the first strand of cDNA. Finally, adaptors were ligated to the fragments, which were used to construct the library and sequenced on an Illumina/Solexa HiSeq2000 platform.

### 2.5. Analysis of RNA-Seq Datasets

After high-throughput sequencing, the high-quality clean data were used for further analyses. All the transcriptome data were uploaded to the NCBI. There were 18 samples of Accession numbers (SRR15291738 to SRR15291755). Furthermore, the Q30, Q20, GC content, and sequence duplication levels of the efficient data were calculated. The pig genome sequence (Sus10.2) and annotation files were downloaded from the Ensemble database [20]. After quality control analysis of the original sequencing dataset, the RNA-seq sequences were compared with the pig genome using TOPHAT and TAIR10 annotations as references [21,22]. Using the transcriptome assembled by Trinity as the reference sequence, the clean reads of each sample were aligned back to the reference sequence [23]. The sequencing output data were analyzed for a single sample, and Cufflinks software was used to calculate the fragments per kilobase of transcript per million mapped reads (FPKM) and the log2FC values of each unigene expression in each sample [24,25]. FPKM represents the number of aligned transcripts per thousand bases per million aligned fragments.

### 2.6. Differential Expression Analysis

This experiment classified the different genes expressed in the three breeds of pigs, compared them in the different samples, and conducted a correlation analysis for the different biological replicates. FPKM was used to calculate the gene expression, which could eliminate the influence of gene length and sequencing data quality on epigenetic expression. After regular logarithmic conversion, the sample data were used for principal component analysis (PCA). The R package gmodels (http://www.r-project.org/ (20 April 2019)) was used to perform the PCA. A weighted gene co-expression network analysis software package in R language was selected to build a co-expression network. The co-expression network was used as shown in the heatmap [26]. For analysis of RNA-seq datasets, the Deseq R software package (1.10.1) was used to analyze the differences in gene expression [27,28]. To control the error discovery rate, the Benjamini-Hochberg method was used to adjust the *p*-value. Gene expression was normalized with a fold change greater than 2 and a significance *p*-value less than 0.05. A Benjamini-Hochberg adjusted *p*-value/false discovery rate (FDR) less than 0.05 indicated differentially expressed genes [29].

### 2.7. Quantitative Reverse Transcription-Polymerase Chain Reaction

SuperScript III (Invitrogen) and Oligo (dT) primers were used to reverse transcribe a total of 1–2 µg of DNase I (RNeasy mini kit)-treated RNA. The synthesized cDNA was subjected to quantitative PCR analysis using the SYBR Premix Ex Taq (Takara) in a Bio-Rad CFX96 real-time PCR system [19,30]. All qRT-PCR reactions were performed for three replications in each cDNA sample, and all the groups were examined as independent measurements to adequately support statistics in the experiments [31]. The Livak method (2-∆∆CT method) was used to calculate the fold change compared to the Yorkshire pig group. All primer sequences used for qRT-PCR are listed in Table 1. 

### 2.8. Wnt and BMP for Hair Growth in Min Pig

Salinomycin sodium salt (Synonyms) and LDN-212854 are inhibitors that exhibit high selectivity for Wnt and BMP, respectively [32,33]. All the inhibitors were dissolved in sunflower oil, which was also used in the control group. The concentrations of Synonyms and LDN-212854 were resolved at 2.5 mg/mL and 5 mg/mL in sunflower seed oil for the cutaneous application, respectively, which is acceptable for external use on the skin, as described previously [34,35]. The Min pigs were shaved dorsally in the anagen period of 5 months for males and females in the cold season. Vehicle control and test compounds were topically applied on the shaved skin every other day for the 3-week duration of the experiments. Skin pigmentation and hair growth were monitored and documented, and the samples were dissected for the next experiments, including qRT-PCR and immunofluorescence, with the experimenter(s) being blind to the treatment conditions. All these experiments were repeated more than six times for male and female Min pigs. 

### 2.9. Immunofluorescence

The freezing tissue slices were washed thrice with 0.01 M PBS and fixed with 4% paraformaldehyde at RT for 15 min. 0.4% Triton (Triton X-100) in the PBS was used to treat and permeabilize the section slides for 2 h at room temperature (RT). The section slides were blocked with 0.3% bovine serum albumin in PBS for 20 min and incubated for 45 min with the specific antibody, Lgr5, at RT. The secondary antibody, goat anti-rabbit fluorescein isothiocyanate isomer (FITC) (Abcam, Cambridge, UK), was incubated with the section slides for 30 min at RT. Finally, the samples were washed and examined using a Zeiss Axioplan2 microscope (Carl Zeiss, Oberkochen, Germany).

### 2.10. Statistical Analysis

All the samples were performed for three replications, and all the groups were examined as independent measurements to adequately support statistics in the experiments. The average duration of recovery and colonization over time were compared between groups using repeated-measure analysis of variance with Bonferroni’s correction using the SPSS software 25.0 (IBM). Statistically significant effects (*p* < 0.05) were further analyzed, and mean values were compared with Tukey’s honestly significant difference test.

## 3. Results

### 3.1. Observation of the Hair of the Three Pig Breeds

**Figure 1:**
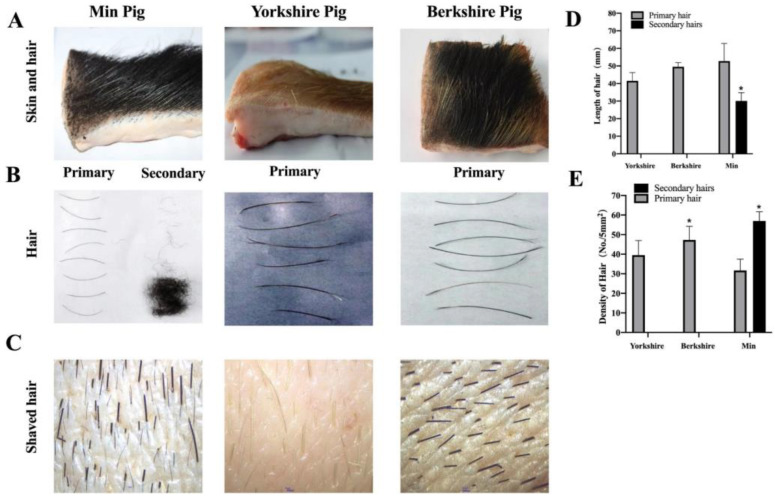
The hair phenotype for three breeds of Min pig, Yorkshire pig, and Berkshire pig. (**A**) Skin and hair phenotypic observation for three breeds. (**B**) Primary hair and secondary hair phenotypic observation for three breeds pigs. (**C**) The shaved hair of three breeds pig were observed with the stereoscopic microscope. (**D**) The length of primary hairs and secondary hairs were calculated from three breeds. (**E**) The number of primary hairs and secondary hairs were analyzed for each breed in 5 mm^2^. * *p* < 0.05.

The hair phenotypes of the three pig breeds, Min, Yorkshire, and Berkshire pigs, are shown in Figure 1A. As shown in Figure 1A, the black primary hairs of the Min pigs were sparse and the skin surface was covered with black secondary hair. The skin of the Yorkshire pigs showed only sparse primary hairs. The skin of the Berkshire pigs had black and white primary hairs. As shown in Figure 1B, the primary and secondary hair lengths of the three breeds were analyzed using a Vernier caliper. There were no secondary hairs in the Yorkshire and Berkshire pigs; however, the length of the secondary hairs in Min pigs was approximately 35.5 mm. As shown in Figure 1C, sections of Min pig skin showed follicle growth patterns and follicle density. As shown in Figure 1D, the Min pig had the longest primary hairs compared to the Yorkshire and Berkshire pigs. The primary hairs of the Berkshire pigs were longer than those of the Yorkshire pigs. The difference in primary hair length was significant in different pig breeds (*p* < 0.05). As shown in Figure 1E, the primary and secondary hair densities of Min, Yorkshire, and Berkshire pigs were analyzed. There were no secondary hairs in the Yorkshire and Berkshire pigs. The average secondary hair density of the Min pigs reached approximately 57 in a 5 mm^2^ area. The number of primary hairs from the Min pigs was lowest compared with the Yorkshire and Berkshire pigs (*p* < 0.05), and the number of primary hairs from the Berkshire pigs was higher than that from the Yorkshire pigs. These results also show that the total number of primary and secondary hairs in the black breed, such as Min pig and Berkshire pig, would have a much higher density than that in the white breed of Yorkshire pig. 

### 3.2. Histological Analysis of Skin and Follicle Morphology

**Figure 2:**
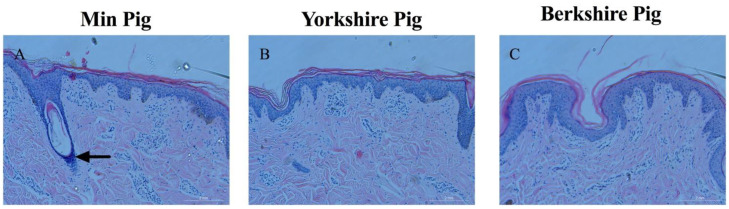
The hair follicle of primary hair and secondary hair samples were studied from the Min pig, Berkshire pig, and Yorkshire pig for histological analysis. Tissue sections of the hair follicles of Min pigs, Yorkshire pigs, and Berkshire pigs in (**A**–**C**). (**A**) The secondary hair follicles of Min pigs with the outer root sheath cells. (**B**,**C**) The secondary hair follicle of Yorkshire pigs and Berkshire pigs did not develop under the skin, which indicates that the hair follicle is in the telogen phase. The magnification of the microscope was 200×.

Hair follicle structure was observed in the Min, Yorkshire, and Berkshire pigs (Figure 2). Under high magnification, the outer root sheath cells were observed (Figure 2A), indicating that the secondary hair follicles were in the anagen phase. The hair follicles of the Berkshire (Figure 2C) and Yorkshire pigs (Figure 2B) were sparse and there was no secondary hair in these two breeds of pig, and these results were the same as the phenotype for the skin and hairs of Berkshire and Yorkshire pigs in Figure 1. Overall, the Min pigs could grow out the secondary hairs whereas the Berkshire and Yorkshire pigs do not have secondary hairs, which was the major difference in hair phenotypes in these three pigs.

### 3.3. Differentially Expressed Genes

**Figure 3:**
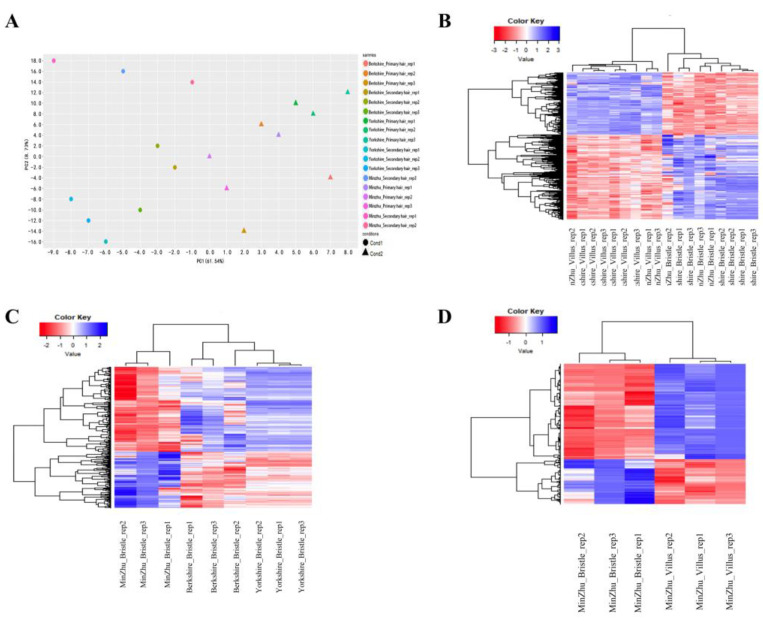
The study compared the gene expression in the primary hair and hair follicle tissues from three pig breeds with high-throughput sequencing. Hierarchical cluster analysis of gene expression based on mRNA sequencing data. A Principal component analysis of gene expression in skin and follicle tissues in three breeds. (**A**) The PCA clusterization successfully separated hair follicle tissues from primary hair and secondary hair samples, and the samples are plotted across the two most variable components (PC 1and PC 2). Cond 1 and Cond 2 represent the secondary and primary hairs, respectively. (**B**,**D**) Gene clustering is rather based on primary hair and secondary hair tissues. The expression values for each gene are arranged in the heat map. (**B**) The primary hair and hair follicle tissues from the Min pig, Yorkshire pig, and Berkshire pig were compared with the gene expression. (**C**) The primary hair follicle tissues from the Min pig, Yorkshire pig, and Berkshire pig were compared with the gene expression. (**D**) The primary hair and hair follicle tissues of Min pigs were compared with the gene expression. Blue indicates the genes with greater expression, and red indicates the genes with lower expression.

The study compared the gene expression in the primary and secondary hair follicle tissues from three pig breeds, which had good consistency in gene expression in each sample from the same group and across the same breeds. The results showed the clustering of gene expression in the skin and follicle tissues from Min, Yorkshire, and Berkshire pigs (Figure 3A). PCA clustering successfully separated hair tissues from the primary and secondary hair follicles in the three breeds (Figure 3A), which showed the significant difference and consistency between the different groups of samples. The generalized linear model was applied to identify gene expression in the primary and secondary hair follicle tissues from the Min, Yorkshire, and Berkshire pigs (Figure 3B). The expression of a total of 547 genes was significantly upregulated in the hair follicle and primary hair tissues. The results showed that 194 genes were significantly differentially expressed in the primary hairs of Min, Yorkshire, and Berkshire pigs, as indicated in the heat map (Figure 3C). The differences were very significant in the Min pigs compared with the Yorkshire and Berkshire pigs. The gene expression in the Berkshire pigs showed similarities to that in the Yorkshire and Min pigs, and the similarities compared with the Yorkshire pigs were more significant than those with the Min pigs. The comparison of the gene for the different expression between the secondary hair follicle and primary hair tissues of Min pigs was analyzed in the heat maps (Figure 3D). There were 194 genes in the secondary hair follicle tissues that were significantly differentially expressed compared with those of the primary hair tissues of Min pigs. As shown in Figure 3D, the difference in gene expression was significant between the secondary hair follicle and the primary hair tissues of Min pigs.

### 3.4. Quantitative Real-Time RT-PCR for the Gene Expression 

**Figure 4:**
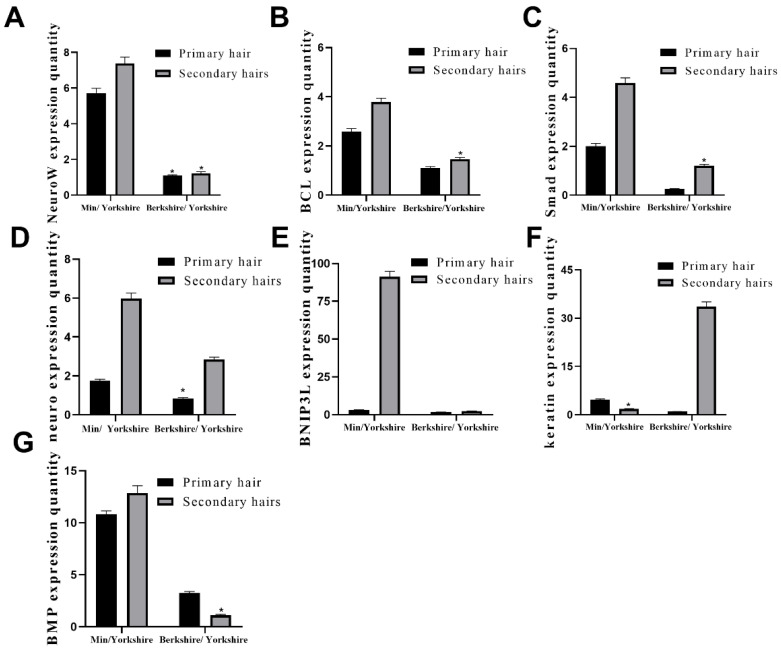
The 7 differentially expressed genes (NeuroW, BCL, Smad, neuro, BNIP3L, keratin, BMP) were selected for the analysis of revalidation with the qRT-PCR in the primary hair and hair follicle tissues from three breeds (**A**–**G**). The GAPDH was used as a reference gene to normalize q-PCR data. Bars represent the standard and error. * *p* < 0.05.

To verify the results of transcriptomic analysis, the seven differentially expressed genes (NeuroW, BCL, Smad, neuro, BNIP3L, keratin, and BMP) were selected for revalidation in the primary hair and hair follicle tissues from the three breeds using qRT-PCR (Figure 4). The Yorkshire pig was used as the control group, which was compared with the Min and Berkshire pigs. The qRT-PCR results confirmed that five genes were significantly differentially expressed between the Min and Berkshire pigs in the primary and secondary hair follicle tissues. There was a significant change in the gene expression in these seven genes. Both primary and secondary hair follicle increased the gene expression in these four genes in the Min pigs compared with the Berkshire pigs, such as NeuroW, Smad, neuro, and BMP (Figure 4A,C,D,G). The expression of BCL and BNIP3L (Figure 4B,E) were only increased in the primary hair follicle from the Min pig, which was higher than the Berkshire pigs at the mRNA level. The keratin expression (Figure 4F) in the Berkshire pigs was the highest compared with that of the primary and secondary hair follicle tissues of the Min pigs. The qRT-PCR results were consistent with the RNA-Seq results. This demonstrates the reliability of the data.

### 3.5. Density of the Follicles under the Wnt and BMP Pathways

**Figure 5:**
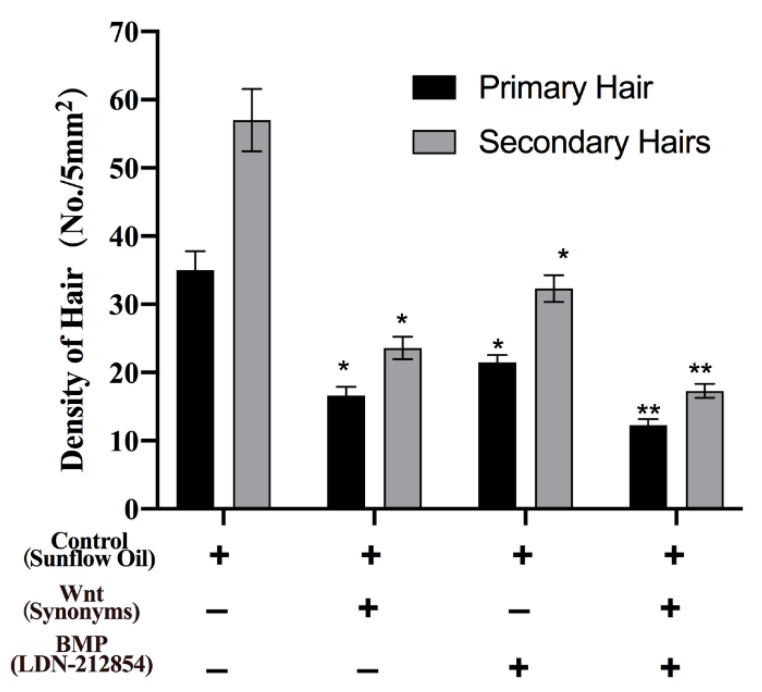
The hair follicle growth under the antagonist of Wnt and BMP were analyzed with the phenotype of hair growth, and both primary hairs and secondary hairs were calculated in the hair follicle stem cells with the pathways for Min pigs. The phenotype of the secondary hairs of Min pigs under stereomicroscope and the number of primary hairs and secondary hairs were analyzed for each breed in 5 mm^2^. Bars represent the standard and error. ** *p* < 0.01, * *p* < 0.05.

To determine the effects of the Wnt and BMP pathways on hair follicle growth, we examined hair growth in the hair follicle stem cells with the antagonist of Wnt and BMP pathways in Min pigs. As shown in Figure 5, the number of hair follicles was higher in the control group than in the Wnt antagonist salinomycin sodium salt-treated group, the BMP antagonist LDN-212854-treated group, and both the Wnt-BMP antagonist-treated groups. The number of hair follicles in the Wnt antagonist group was lower than that in the BMP antagonist group. The number of hair follicles in the Wnt-BMP antagonist group was the lowest compared with that of the Wnt antagonist, BMP antagonist, and control groups. The results indicate that Wnt and BMP can stimulate the growth of hair follicles, and the effect of Wnt was better than that of BMP in the growth of hair follicles in Min pigs. Both Wnt and BMP functioned in stimulating the growth of primary and secondary hairs in the Min pigs. 

### 3.6. Wnt and BMP Signaling Molecules Stimulate the Hair Follicle Stem Cell Differentiation

**Figure 6:**
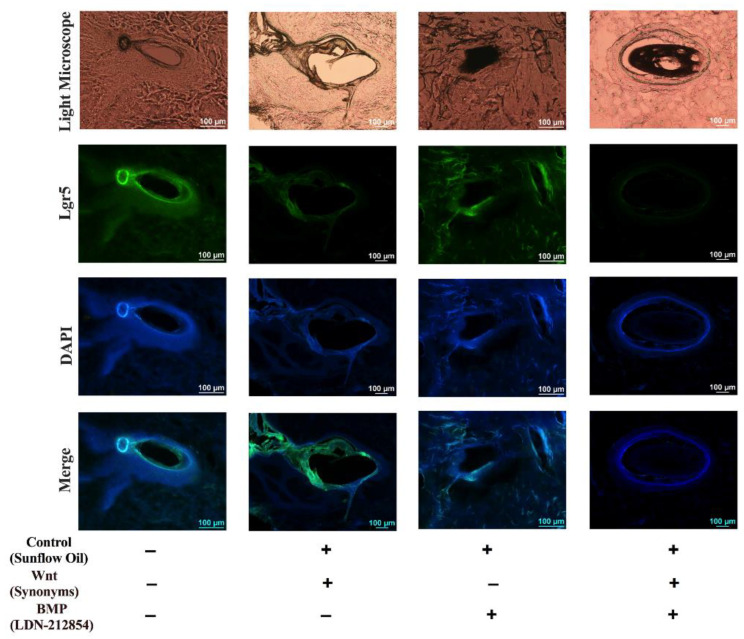
The indirect immunofluorescence was used to study the follicle stem cells with the marker of Lgr5 in the hair follicle of frozen section from Min pig. The Wnt and BMP singles play the role in the inhibition of the hair follicle stem cells, which cause the fail to induce follicle growth.

The effects of the Wnt and BMP pathways on hair follicle growth were studied. Hair follicle stem cells were studied with the marker, Lgr5, in the hair follicles of the frozen sections from Min pigs. It is common knowledge that the inhibition of hair follicle stem cells can cause failure to induce follicle growth. The antagonists Wnt and BMP were used to study hair follicle stem cells. We analyzed the expression of genes in hair follicle stem cells from Min pigs using immunofluorescence staining. The antagonisms were resolved with sunflower oil and the samples served as the control group in the study. The protein level of Lgr5 in the hair follicle stem cells was the highest in the control group of sunflower oil. The results (Figure 6) indicated that the protein levels of Lgr5 in the BMP and Wnt groups decreased in the hair follicle stem cells compared with that of the control group. The number of hair follicle stem cells in both the Wnt and BMP antagonist groups decreased significantly compared to that of the control group. The protein level of Lgr5 in the Wnt antagonist group was lower than that in the BMP antagonist group. The protein level of Lgr5 in the Wnt-BMP antagonist group was the lowest among the four groups. All these results confirmed the phenotype of hair follicle growth.

### 3.7. Quantitative Real-Time RT-PCR Analysis Results

**Figure 7:**
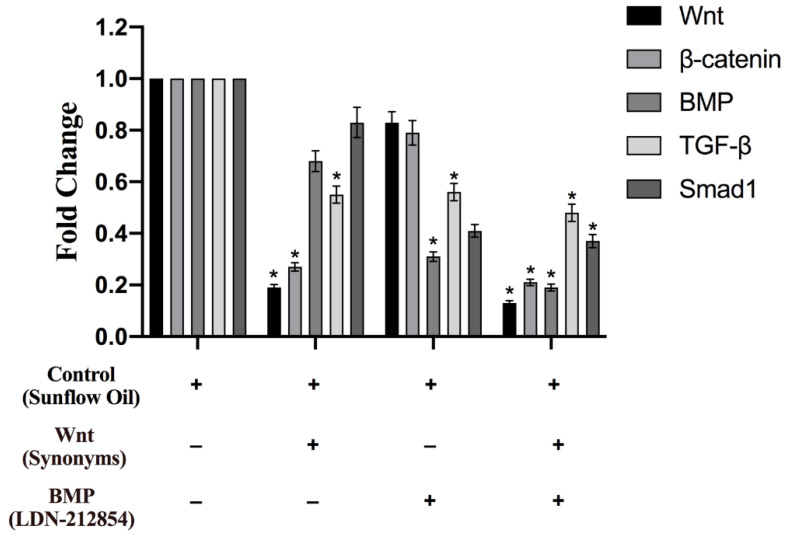
The qRT-PCR analyzed the relative gene expression under the Wnt and BMP singles, which were blocked with the antagonist of Wnt and BMP. The GAPDH was used as a reference gene to normalize q-PCR data. Bars represent the standard and error, * *p* < 0.05.

As shown in Figure 7, the result of qRT-PCR indicated that there was a significant difference in the expression of the Wnt signaling pathway in hair follicle stem cells in the synonym group compared with that in the control group. The Wnt/β-catenin antagonist of synonyms was effective in inhibiting the expression of relative genes from these signaling pathways, especially Wnt, which showed that the effective inhibitor synonyms of signal transduction could reduce relative gene expression in the signaling pathway, decreasing the Wnt expression. The hair follicle stem cell signaling pathways of BMP, TGF-β, and Smad1 were also examined, which was the downstream signal in the transduction pathways Wnt and BMP. The gene expression of β-catenin and TGF-β were significantly decreased compared with the inhibition of Wnt. The BMP inhibitor, LDN-212854, was added to analyze its role in the signal transduction pathways of hair follicle stem cells. The expression of the TGF-β signaling pathway was also significantly decreased in the LDN-212854 group compared with that of the synonym group. However, the relative expression of Smad1 was also significantly lower than that in the synonym and control groups, which indicated that the inhibitor, LDN-212854, could also inhibit the expression of the relative genes in the signaling pathway. Most importantly, the expression of the hair follicle stem cell signaling pathway in the group with both synonym and LDN-212854 was lower than that in the other three groups, which indicated that both the synonym and LDN-212854 were coordinative in the inhibition of the hair follicle stem cell signaling pathway. Based on these experimental results, we could infer that the Wnt and BMP signaling could stimulate follicle stem cells.

## 4. Discussion

Chinese pig breeds were Chinese local breeds, which have many different characteristics than European pig breeds. Therefore, these different phenotypes of hair from different pig breeds may be based on their genetic inheritance. To date, most studies related to the hair follicle development of domestic animals have focused on two aspects, namely, morphology and molecular genetics [7,36,37]. The structure and morphology of hairs, such as primary and secondary hairs, from Min pigs are different than those of lean-type pigs, such as Yorkshire pigs and Berkshire pigs, which do not grow secondary hairs. Historically, the Berkshire pig has a blood relationship with the Meishan pig, a breed of pig in China; therefore, the coating thickness of the Berkshire pig is between that of the Min and Yorkshire pigs [38,39]. Researchers have found that during skin follicle development in the Australian cashmere goat, all primary follicles were present but only a few secondary follicles were mature at birth, and the number of secondary follicles increased significantly faster than that of primary follicles [40]. Their study is similar to this one on the Min pig, where the secondary follicles mature with the character of seasonality. Therefore, the development of hair follicles is largely influenced by genetics. With the changes in the environment of animals in cold weather, the animals have specific characteristics that protect their functions in such environmental conditions, such as thicker coats and secondary hair, which reduces heat loss from the animal and cold skin irritation [41]. The results of a study revealed that the differences in hair growth rate affect both sow parity and the number of piglets [42], and hair growth and length may vary depending on cortisol concentrations caused by downregulation of the hypothalamic-pituitary-adrenal axis [43]. These reproductive performances and behaviors are present in the Min pigs. The color of hair played a role in the growth of hair in our study comparing Min, Yorkshire, and Berkshire pigs, and the black hair would grow much faster than the white hair in the pigs. 

In this study, a direct comparison of the transcriptome dynamics for Min, Yorkshire, and Berkshire pigs with the hair follicle recycling unexpectedly revealed the huge difference between these two different types of hair follicles. The present study compared gene expression in the primary and secondary hair follicle tissues from three pig breeds, which showed a certain consistency in gene expression within each group. There was a significant difference in gene expression between the secondary hair follicles and primary hair tissues of Min pigs. Differentially expressed genes from the transcriptomic analysis of revalidation indicated that there was a correlation in the gene expression in primary hair and hair follicle tissues from the three breeds. A study compared differences in mRNA expression and microRNA expression during the growth and repose stages of cashmere goat skin samples, and it found that hair follicle initiation and development were related to MiR-195 and the gene SMAD2, whilst protein-specific MiR-195 regulated the Wnt/β-catenin pathway in the telogen-anagen hair follicle of the goats [44]. The WNT, BMP, TGF-β, and Hedgehog signaling pathways were found in hair follicle cycling in both cashmere and milk goats with the character of the seasonal development [45].

In this study, the library used for RNA sequencing was selected, and the qRT-PCR revalidation showed that the data were reliable, and could be used for the subsequent analysis and study of the co-expressed gene and differential gene expression in the hair follicles from Min, Berkshire, and Yorkshire pigs. The screened genes also demonstrated the concrete cell signaling mechanisms of primary hair and hair follicle development. High-throughput sequencing analysis results showed that some of the significantly differentially expressed genes were related to the neuronal and developmental signaling pathways in the primary and secondary hairs [46,47,48]. After an in-depth study of the gene expression differences in the three pig breeds, this study found that some signal factors related to hair circulation can promote the proliferation of hair follicle stem cells and the growth of hair follicles [49]. During hair follicle development, the Wnt and BMP signaling pathways can stimulate hair follicle growth [50]. With genetics and chromatin landscaping, researchers have found that the McSCs, BMP, and WNT pathways are stimulated following WNT-mediated activation, thereby triggering the commitment of proliferative progeny [51]. BMP upregulates various signaling pathways, such as Ctnnb1, Lrp6, Bmpr1a, and PTEN, and consequently induces hair cell differentiation, which has been a potential therapeutic target for hair loss and the short-hair phenotype [52,53]. Reactome analysis revealed the strong enrichment of DEGs in the TGF-β signaling pathways in hair follicle samples of milk goats, but not in cashmere goats [44]. Compared with the study of hair follicle cycling in these goats and pigs, we could find that hair follicle cycling was a characteristic of interspecific difference or seasonal effect.

Opinions differ regarding whether the hair growth pattern is controlled by a single molecule of Wnt and BMP from stem cells [50,54]. Our results are the first to show that Min pigs specifically activate the Wnt signaling molecule of the stem cell in their skin with hair growth. Lgr5, which was used in our research on stem cells from Min pigs, was identified as a marker during trafficking through stem cell properties and contributes to hair follicle growth [55]. Damage-activated stem/progenitor cells in the hair follicle play important roles in regenerating lost cells and in tissue repair. Lgr5 is known to stimulate hair follicle development; however, the exact mechanism of the hair cycle is still unclear [56]. The dynamic Wnt in the hair follicles of Min pigs suggests a link between the skin and hair systems. Subcutaneous fat, leptin, and neural and stem cell molecules in hair have a thermoregulatory function in the dermal papilla of hair follicles, which coordinate the function of the skin and hair in response to the external environment and may have implications for the evolution of integuments in Min pigs. This study found that the skin and hair of Min pigs have a specific character in the development mechanisms of secondary hairs, which may have potential applications in laboratory animals and comparative medicine. 

## 5. Conclusions

The national-level protected Min pig has the specific character of secondary hair growth in winter and seasonal cycling, which is different from Yorkshire and Berkshire pigs. The structure and morphology of hairs, such as primary and secondary hairs, from Min pigs are different from those of lean-type pigs, such as Yorkshire pigs and Berkshire pigs, which do not grow secondary hairs. The development of hair follicles is largely influenced by genetics, and a direct comparison of the transcriptome dynamics for Min, Yorkshire, and Berkshire pigs with hair follicle recycling unexpectedly revealed the huge difference between these two different types of hair follicle. Our results are the first to show that the Min pig specifically activates the Wnt signaling molecule of the stem cell with Lgr5 in the skin with hair growth, which was used in our research on stem cells from Min pigs, which was identified as a marker during trafficking through stem cell properties, and which contributes to hair follicle growth. The research on the skin and hair of Min pigs shows the specific character of the development mechanisms of the secondary hairs, which may have potential applications in laboratory animals and comparative medicine.

## Figures and Tables

**Table 1:** Primer sequence for qRT-PCR.

Gene	NCBI Accession	Sequence (5′-3′)
BCL2	XM_001927592.3	F: ATGTTCAGGTCCAAACGCTCGG R: CTGCCCTTGCTCCCATCCTC
SMAD6	XM_003480446.3	F: GCGGCGACTTTGGCGAAGT R: GCGTCCCGGGGCCGCCGCAG
Neuropeptide W	NM_213786.1	F: CCTCCGGAGCCAGTTCCTGG R: AGTAACAGCAATGCCAGCAGCC
keratin	XM_003126159.4	F: CTCACCTATAGCACCACCCC R: GAGAGCAGCGAAGGGTCTTT
BMP	NM_001201485.1	F: CCCAAATTCCCCTCTCACCC R: GCTACCGTCAGGCTGATACC
neuro	NM_001123152.1	F: ATGTCCATCTTGTTTTATAT R: CTGGTAATTTTCCTGAAGGTCC

## Data Availability

Once this manuscript is accepted, the data supporting the results of this study will be made publicly available in any publicly accessible repository.
